# Treatment planning and outcomes effects of reducing the preferred mean esophagus dose for conventionally fractionated non‐small cell lung cancer radiotherapy

**DOI:** 10.1002/acm2.13150

**Published:** 2021-01-25

**Authors:** Ellen D. Yorke, Maria Thor, Daphna Y. Gelblum, Daniel R. Gomez, Andreas Rimner, Narek Shaverdian, Annemarie F. Shepherd, Charles B. Simone, Abraham Wu, Dominique McKnight, Andrew Jackson

**Affiliations:** ^1^ Department of Medical Physics Memorial Sloan Kettering Cancer Center New York City NY USA; ^2^ Department of Radiation Oncology Memorial Sloan Kettering Cancer Center New York City NY USA

**Keywords:** dose‐volume constraints, non‐small cell lung cancer, normal tissue complications probability, treatment planning

## Abstract

Based on an analysis of published literature, our department recently lowered the preferred mean esophagus dose (MED) constraint for conventionally fractionated (2 Gy/fraction in approximately 30 fractions) treatment of locally advanced non‐small cell lung cancer (LA‐NSCLC) with the goal of reducing the incidence of symptomatic acute esophagitis (AE). The goal of the change was to encourage treatment planners to achieve a MED close to 21 Gy while still permitting MED to go up to the previous guideline of 34 Gy in difficult cases. We compared all our suitable LA‐NSCLC patients treated with plans from one year before through one year after the constraint change. The primary endpoint for this study was achievability of the new constraint by the planners; the secondary endpoint was reduction in symptomatic AE. Planners were able to achieve the new constraint in statistically significantly more cases during the year following its explicit implementation than in the year before (*P* = 0.0025). Furthermore, 38% of patients treated after the new constraint developed symptomatic AE during their treatment as opposed to 48% of the patients treated before. This is a clinically desirable endpoint although the observed difference was not statistically significant. A subsequent power calculation suggests that this is due to the relatively small number of patients in the study.

## 1 Introduction

Acute esophagitis (AE) is a common complication in patients treated with conventionally fractionated radiation therapy for locally advanced non‐small cell lung cancer (LA‐NSCLC). The standard prescription dose for LA‐NSCLC at our institution is 2 Gy/fraction for 30–35 fractions. In this often elderly and frail population, swallowing difficulty due to moderate AE can further debilitate the patient and severely impact their quality of life. More severe AE may require treatment breaks, hospitalization, or early treatment termination and may be a risk factor for late esophageal complications and importantly, the development of high grade treatment‐related esophagitis is directly related to overall survival in LA‐NSCLC patients.^1^ Many LA‐NSCLC patients receive concurrent chemotherapy (CCRT), which has been found to increase the frequency and severity of AE in comparison to sequential or no chemotherapy.^2^

To limit normal tissue complications, our institution sets organ at risk (OAR) treatment planning dose constraints for each type of treatment in a departmental spreadsheet and updates them in response to new published data or internal analyses of our own results. The constraints fall into two categories: ‘limits’ which can only be violated after consultation with the prescribing physician and possible peer review by another attending physician, and ‘guidelines’ which, while desirable, can be violated in the interest of target coverage or sparing of other higher priority OARs. Guideline violation is always discussed with the prescribing physician. In all cases, planners aim to both meet disease‐specific target coverage metrics and to minimize normal tissue doses.

For LA‐NSCLC patients, a physician contours the esophagus from the cricoid cartilage to the gastro‐esophageal junction on the planning CT scan. For several years, the esophageal planning guidelines for these patients have been mean esophageal dose (MED) ≤ 34 Gy and V60_Gy_ ≤ 17%; the latter is conditional on not compromising target coverage without explicit physician approval. However, there are several published dose–volume models of symptomatic AE and an analysis based on four such models was performed at our institution as part of a long‐range effort to integrate such a model directly into planning.^3^ The model predictions were compared with the outcomes of Stage IIIA‐IIIB LA‐NSCLC patients treated at our institution with CCRT and IMRT between 2004 and 2014.^3^ The best model was found to be that of Huang et al.^4^, ^5^

The model predicted that AE would be notably reduced if MED could be kept in the low 20 Gy range. In September 2018 (before the publication of Reference 3), we changed our clinical guidelines to state that MED ≤ 21 Gy was preferred. Planners were instructed to aim for a MED below 21 Gy. If that prevented acceptable target coverage, they were asked to aim for a MED between 21 Gy and 34 Gy. The V60_Gy_ guideline remained in place and no ‘limits’ were added. The guidelines and general plan requirements are the same regardless of whether the patient receives CCRT or not.

Each patient has a weekly on‐treatment visit (OTV) with an attending radiation oncologist. Complications noted at the OTV are recorded in the electronic medical record (EMR) per the Common Terminology Criteria for Adverse Events (CTCAE) v4.0.

When the new esophagus planning guideline was instituted, we had two questions:

1. Was the lower MED sufficiently easy to achieve that simply sensitizing planners to the desirability of a lower MED would lead to a significant reduction in the planned MED?
2. If the answer to the first question was ‘yes’, would an accompanying decrease in incidence of symptomatic AE be observed: specifically would the incidence of Grade 2 or higher AE (AE2+) reported in OTVs during the course of treatment be reduced?

## 2 Methods

All patients were planned in Eclipse (v.15; Varian Medical Systems, Palo Alto, CA) for 6 MV photon treatment on a variety of Varian linacs; doses were calculated with the Analytical Anisotropic Algorithm (AAA) algorithm. At simulation, the patient was immobilized in an alpha‐cradle (Smithers Medical Products, Inc.) mounted on a device that was indexed to the couch. Isocenter and associated skin marks (tattoos) were placed at simulation, where both a free‐breathing scan and a 4DCT were acquired. The physician delineated the GTV based on the free‐breathing scan and a fused FDG‐PET scan, determined the internal target volume (ITV) using the 4DCT, formed the clinical target volume (CTV) by adding a 5–7 mm 3‐dimensional margin to the ITV and formed the planning target volume (PTV) by a further 5 mm margin around the CTV. The esophagus, heart, and other OARs that might receive significant dose (e.g. brachial plexus, liver) were delineated by a physician while the planner delineated lungs and the spinal canal. Patients were set up by tattoos followed by daily orthogonal images matched to the spine on an orthogonal pair of digitally reconstructed radiographs (DRRs); a cone‐beam CT was acquired weekly, primarily to follow tumor changes.

We reviewed all consecutive patients treated definitively for LA‐NSCLC with a prescription of 2 Gy/fraction for one year before and one year after the new guideline was put in place. No other planning, technique, delivery, or imaging changes were deliberately introduced during that period. For each patient, we recorded the MED and V60_Gy_ from the treatment plan and whether the patient had CCRT. The clinical charts were retrospectively reviewed to determine the maximum grade of esophagitis noted in at least one weekly OTV. Esophagitis after the treatment course was finished was not recorded because of the sporadic nature of the post‐treatment records in the EMR. The analyses comparing clinical and dosimetric factors before and after the guideline change was done with Fisher’s test and significance was determined by *P* < 0.05.

As part of our analysis, we examined agreement of outcome with the model on which the guideline change was based.^3^, ^4^ This predicts the percent of patients with CTCAE4 v4.0 Grade 2 or higher AE (AE2+) to be given by:

(1) $$\%\text{AE2}+\;=100/\left(1+e^{‐X}\right)$$

where

X = 1.5 CCRT + 0.07 MED −3.1.

The variable CCRT is 1 for patients with concurrent chemotherapy and 0 for those without; MED is the mean esophagus dose in Gy. The model was applied to Groups A and B with no re‐fitting performed. Model discrimination was assessed through the area under the receiver‐operating characteristics curve (AUC) and calibration between observed and predicted AE2 was judged visually.

This study was completed under an institutional review board approved protocol.

## 3 Results

Sixty‐four patients meeting criteria were treated the year before (Group A) the guideline change and coincidentally 64 were treated the year after (Group B). The results for the two groups that is described below are also summarized in Table 1.

**Table 1:** Groups A (within 1 year before guideline change) and B (within 1 year after) characteristics.

	Subcategories	Group A	Group B
Number of patients		64	64
Males (%)		36 (56.3%)	33 (51.6%)
Age (y): Median, [range]		67, [36–89]	69, [48–86]
Planned fractions,: Median [range]		30, [27–35]	30, [30–36]
Plan types		3DCRT: 2 Fixed Gantry IMRT: 44 VMAT: 18	Fixed Gantry IMRT: 39 VMAT: 25
CCRT: number (%)		47 (73%)	51 (80%)
PTV (cc): Median, [range]		530.5, [104–1380.1]	440.5, [85.5–1104.6]
MED (Gy): Median, [range]		25.6, [5.2–40.5]	20.2, [4.5–33.9]
Esophagus V60 (%): Median, [range]		11.4, [0–51.3]	5.95, [0–30.2]
MED < 21 Gy number (%)		21 (32.8%)	39 (60.9%)
	MED < 21 Gy AND CCRT: number (% with CCRT)	13 (27.7%)	30 (58.8%)
AE2+ number (%)		31 (48.4%)	24 (37.5%)
	AE2+ AND CCRT : number (% of all pts in group with CCRT)	27 (57.4%)	21 (41.2%)
AE2+ and MED < 21 Gy: number (% of all patients in cohort)		3 (4.7%)	13 (20.3%)
AE2 and MED > 21 Gy; number (% of all patients in cohort)		28 (43.8%)	11 (17.2%)

CCRT, Concurrent chemotherapy; MED, mean esophagus dose; AE2+, Grade 2 or higher acute esophagitis.

Group A had 36 males and 28 females; Group B had 33 males and 31 females. The median age in Group A was 67 y [range 36–89] and in Group B was 69 y [range 48–86]. All patients were treated with 2 0 Gy/fraction. The median number of planned fractions in Group A was 30 [range 27–35; specifically 52 with 30 fractions, 4 with 35, 6 with 33 and one each with 29 and 27] and was also 30 in Group B [range 30–35; specifically 55 30 fractions, 3 of 35 fractions, 5 of 33 and one of 32]. The plan types in Group A were 2 3DCRT, 44 sliding window IMRT and 18 VMAT, while in Group B they were 39 IMRT and 25 VMAT. The plan types were not significantly different between the two groups (*P* = 0.26, considering 3DCRT and IMRT, for both of which beam angle selection is a critical factor, as the same plan type). In Group A, 47 patients (73%) had CCRT; in Group B, 51 (80%) had CCRT. Similarly this difference was not significant (*P* = 0.53).

**Fig. 1:**
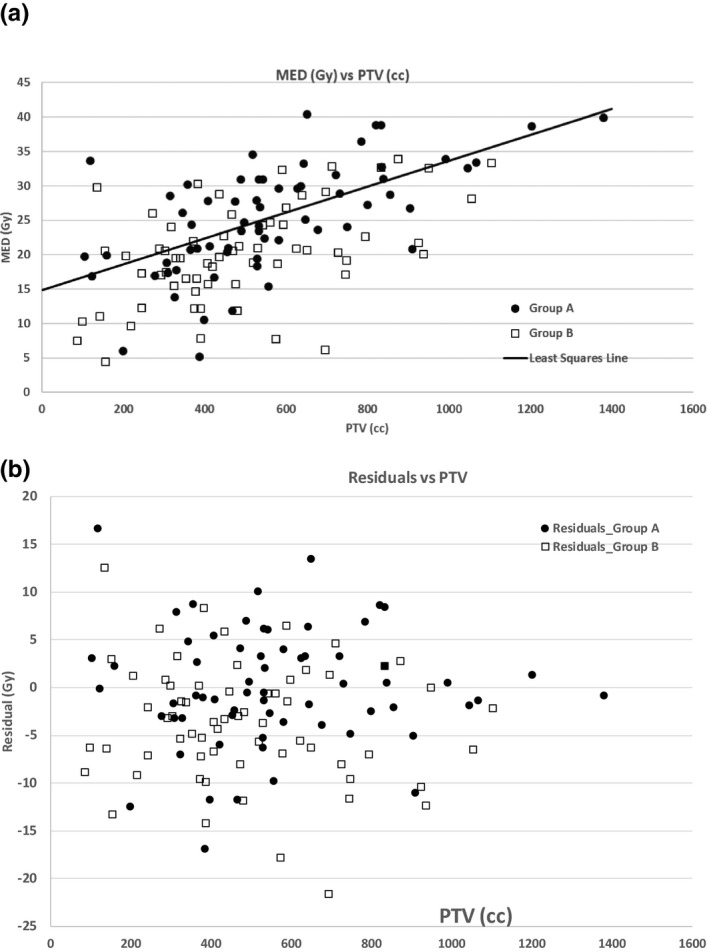
(a) The distribution of MED versus the PTV for Groups A and B. The solid line is the least‐squares fit for Group A (Rsq = 0.38). (b) Residuals (actual MED minus MED predicted by linear fit to Group A MED versus PTV) for groups A and B. Group A is well‐described by the linear fit so Group A data points are approximately equally disposed around zero. The Group B data points are displaced toward negative residuals, demonstrating the effect of the tighter MED guideline over and above effects of PTV differences in the two groups.

The median and range of the PTV for Group A are 530.5 cc [104–1380 cc] and for Group B are 440.5 cc [85.5–1104.6 cc]. Other factors, such as PTV location relative to esophagus, might also have an impact but because there was no deliberate change in patient selection during the study time interval, we did not investigate this. All OAR constraints (primarily lung_minus_GTV, cord and heart) were met for all plans and clinical metrics of plan coverage were similar between the groups. For example, as a percent of prescription dose, the mean ± standard deviation of the PTV D95 in groups A and B, respectively, are 99.4 ± 2.9 and 99.6 ± 2.2 while for the minimum PTV dose they are 80.6 ± 12.3 and 79.4 ± 13. The median and range of MED and esophagus V60_Gy_ for Group A are 25.6 Gy [5.2–40.5 Gy] and 11.4% [0–51.3 %] while for Group B they are 20.2 Gy [4.5–33.9 Gy] and 5.95% [0–30.2%]. While lower esophagus doses, as represented by V5, are not among our planning constraints, we observed that V5 is moderately correlated with MED (Rsq = 0.68 for Group A and 0.60 for Group B) and that V5 is higher for Group A (mean ± SD = 62.1% ± 16.1%) than for Group B (56.6% ± 14.2%). MED is moderately correlated with both PTV (Group A Rsq = 0.38, Group B Rsq = 0.26) and esophagus V60 (Group A Rsq = 0.31, Group B Rsq = 0.41). A scatter plot of MED versus PTV for the two groups along with the linear least‐squares fit line to the Group A data show that the Group B MEDs are systematically low [Fig. 1(a)]. To disentangle the MED reduction caused by the smaller PTVs in Group B from the effect of the treatment planning guideline change, we calculated the residuals – the difference between the observed MEDs and the MED predicted by the Group A least‐squares fit line [Fig. 1(b)]. Upon applying the Wilcoxon rank sum test, we find that the rank sum of the residuals is significantly smaller for Group B (*P* = 0.0007), and thus the null hypothesis‐ that the difference in MED is entirely explained by the lower PTVs of Group B – can be rejected.

Hereafter, we focus on the relationship between the incidence of AE2+ and MED, which was the subject of the stated constraint change.

In Group A, the MED was less than 21 Gy for 21 patients (33%) of whom 13 had CCRT (28 % of Group A CCRT patients). In Group B, the MED was less than 21 Gy for 39 patients (61% ) of whom 30 had CCRT (59% of Group B CCRT patients ). The MED dosimetric difference between the two groups was significant (*P* = 0.0025 for all patients, 0.0023 for those with CCRT).

**Fig. 2:**
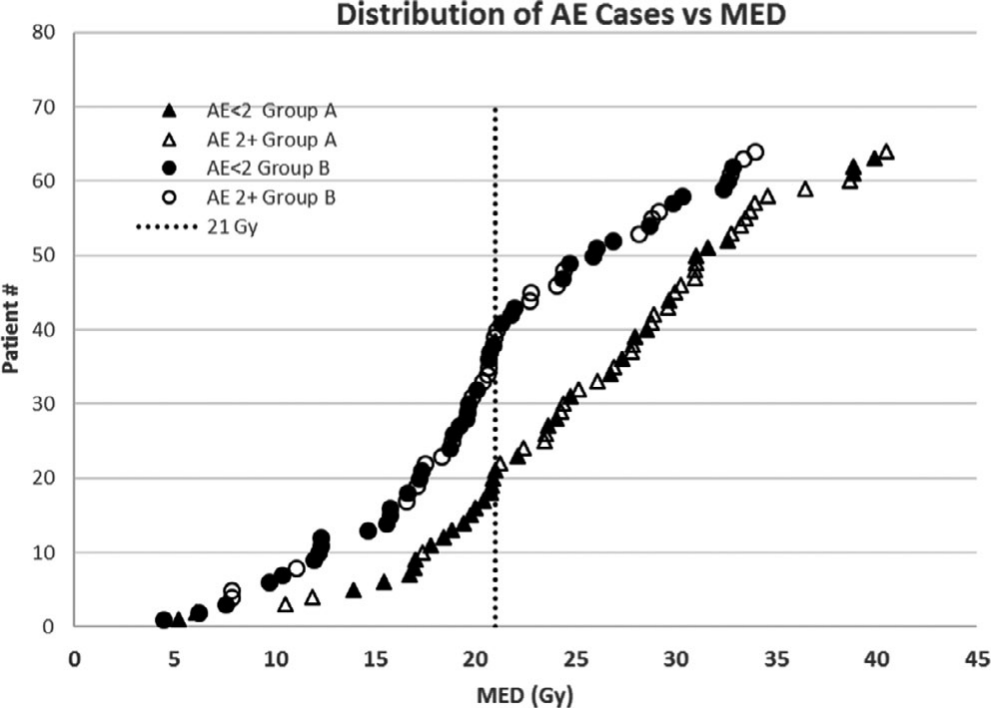
Distribution of AE less than or ≥ Grade 2 ranked according to MED for the two groups. The vertical line is 21 Gy.

**Fig. 3:**
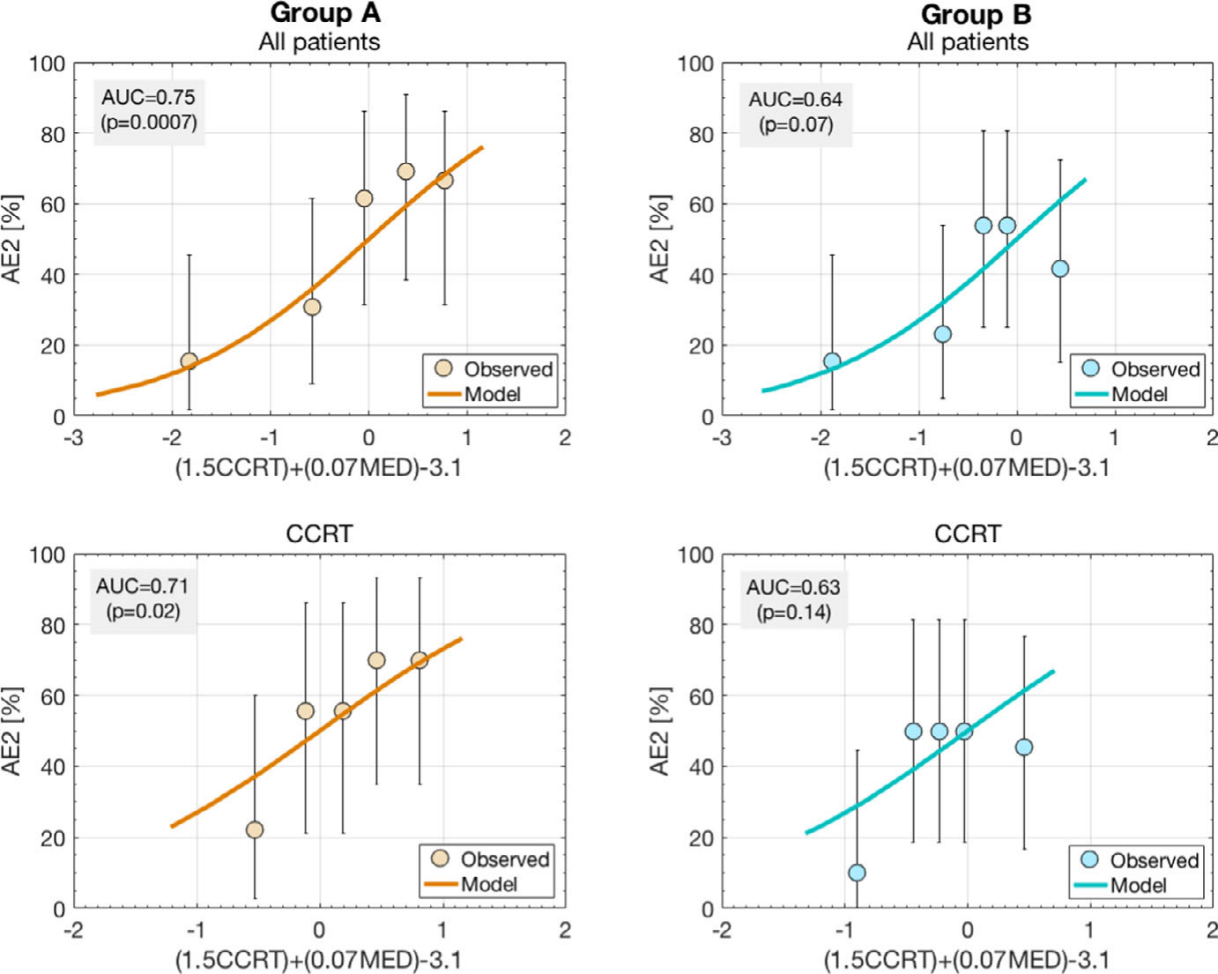
Agreement between the data (in quintiles) and the acute esophagitis model (Reference 3) on which the guideline change is based. AUC refers to the area under the curve in the receiver‐operating characteristics curves for the groups described in each figure’s title.

The maximum grade of AE noted in the weekly OTV records was as follows: in Group A, 31 patients (48%) had AE 2+ (Grade 3 for 2, Grade 2 for 29) while in Group B, 24 patients (38%) had AE2+ (24 Grade 2). Of patients with AE2+, the MED was less than 21 Gy for three patients in Group A (14% of patients with MED < 21 Gy) and for 13 patients in Group B (33% of patients with MED < 21 Gy). Of the AE2+ cases in Group A, 27 had CCRT (57% of the Group A patients with CCRT had AE2+); of the AE2 patients in Group B, 21 had CCRT (41% Group B patients with CCRT had AE2). The association with CCRT was significant for Group A but not Group B (*P* = 0.02 and 0.34 respectively). Figure 2 shows the distribution of AE2+ cases in the two groups ranked by MED. It can also be seen in this figure that the overall distribution of AE2+ cases in Groups A and B with respect to MEDs is qualitatively different. Specifically there are 70% more patients with MED between 18 and 21 Gy in Group B than in Group A(17 Group B, 10 Group A). However, as shown in Fig. 3, the model from which the new guideline was derived^3^ described the AE rates both before and after the new guideline. Though discrimination dropped slightly in the Group B subset (AUC = 0.75 vs. AUC = 0.64), overall the predicted AE rates agreed well with observed rates.

Although the number of patients with AE2+ was almost 30% larger in Group A (31 cases, 48% of the group) than in Group B (24 cases, 38% of the group), this did not translate to a significant difference in AE2+ between either the two cohorts (*P* = 0.28) or only those with CCRT (*P* = 0.16). The difference in the distributions of AE2+ with MED was significant (*P* = 0.0007 for all patients, 0.0011 for those with CCRT), which is associated with the previously remarked distribution change. Nonetheless, when combining the two cohorts, significantly more patients had AE2+ if their planned MED was above 21 Gy than if it was below. The p‐values are 0.0006 for all cases and 0.001 for only cases with CCRT.

**Fig. 4:**
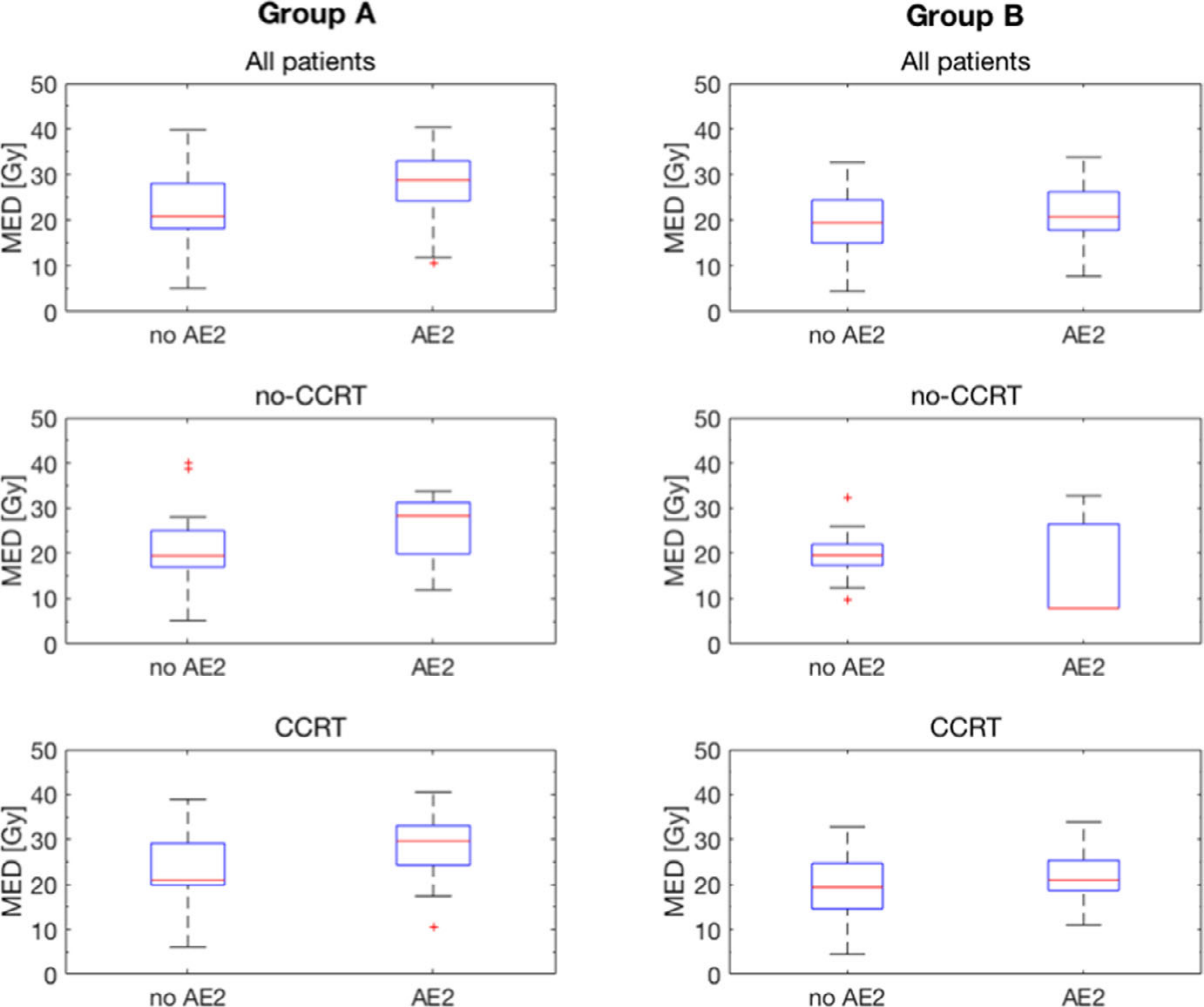
Graphical summary of differences between Groups A (before guideline change) and B (after change).

The gross differences between Groups A and B before and after the change in planning guideline are shown graphically in Fig. 4. Details of the features that were explored and their statistical significance are summarized in Table 2.

**Table 2:** Patient and plan features investigated and their statistical significance.

Factor being considered	Fisher's test *P*‐value	% Group A	% Group B
Difference in use of CCRT between A and B cohorts	0.53	73	80
Difference in AE2+ incidence between Groups A and B_ all pts	0.28	48.4	37.5
Difference in AE2+ incidence between Groups A and B_Patients with Chemo‐RT	0.16	57.4	41.2
Difference in MED ≤ 21 Gy between A and B	**0.003**	**32.8**	**60.9**
Difference in AE2+ between cohorts for MED ≤ 21 Gy vs > 21 Gy; all pts in cohort	**0.0007**	**14.3% < 21 Gy** **65.1% >21 Gy**	**65.1%<21 Gy** **44% >21 Gy**
Difference in AE2+ between cohorts for MED ≤ 21 Gy AND CCRT vs. > 21 Gy; AND CCRT	**0.001**	**15.4% < 21 Gy** **78.1% > 21 Gy**	**36.7% < 21 Gy** **41.7% > 21 Gy**
		**Groups A and B Combined**	
Incidence of AE2+ for patients with CCRT and MED ≤ 21 Gy v.s > 21 Gy; combined groups	**0.001**	**34.9% for MED < 21 Gy** **72.7% for MED > 21 Gy**	
Incidence of AE2+ for MED ≤ 21 Gy vs > 21 Gy; combined groups: all patients	**0.0006**	**26.7% for MED < 21 Gy** **57.4% for MED > 21 Gy**	

The significant entries are bolded.

CCRT, Concurrent chemotherapy; AE2+, Grade 2 or higher acute esophagitis; MED, mean esophagus dose.

## 4 Discussion

Radiation therapy treatments must strike a balance between effective tumor control and avoidance of serious treatment complications. To do this, treatment planners must avoid ‘cold spots’ in the target while respecting normal tissue dose‐distribution constraints, which are often based on a combination of local clinical experience and peer‐reviewed, published studies. If the OAR constraints are not achievable for a specific patient, the physician may lower the prescription dose, accept reduced target coverage or accept a higher risk of toxicity. Although IMRT and VMAT plans are ‘optimized’, most optimization algorithms penalize constraint violations but do not reward doing better. When we introduced a lower mean esophagus dose constraint (prefer MED ≤ 21 Gy) for conventionally fractionated LA‐NSCLC treatment plans based on a published model, we did not eliminate the original higher constraint (34 Gy) because we did not know how often planners would be able to achieve satisfactory coverage together with the desired lower MED. The results of this study show that planners were indeed able to do better; 61% of the Group B plans had MED below 21 Gy as opposed to 33% of the Group A plans, and this difference was statistically significant.

Figure 3 shows that the dependence of our observed symptomatic acute esophageal toxicity on MED agrees with the model that prompted the change. As expected from the model, in both the combination of Groups A and B and in the combination of Group A and B patients with CCRT, the number of AE2+ cases with MED below 21 Gy was statistically significantly lower than for those with MED above. The incidence of AE2+ in Group B was 38% while in the earlier Group A it was 48%; for patients with CCRT, the AE2+ rates were 41% in Group B versus 57% in the earlier Group A. Though the changes in this relatively small study are not statistically significant, we are encouraged by both the improvement in outcome and the agreement with the model (Fig. 3).

The small number of patients is a limitation of this study. Therefore, we performed a power calculation to determine the number of patients in the two groups that would be needed to detect, with *P* ≤ 0.05, the observed change in AE2+ due to the changed MED constraint. To detect this with 80% power would require a total of at least 285 patients with CCRT or at least 638 patients for all patients, regardless of CCRT status. To detect it with 90% power would require a total of at least 379 patients with CCRT (848 patients regardless of CCRT).

A further limitation is that the esophagitis grade noted at each OTV is, to some extent, subjective as it relies on a physician’s interpretation of the conversation with the patient and the patient’s tolerance for discomfort. Because of the variability of post‐treatment records in our EMR, we did not consider esophagitis after the treatment course was finished. To our knowledge, there is no study of esophagitis relating the esophageal dose accumulated at the time of initial AE2+ diagnosis in each patient. Such a study is beyond the scope of this paper. However, in a larger group of patients (perhaps involving imaging biomarkers as well as clinical records,) it might allow us to understand how accumulation of tissue damage results in clinical esophagitis.

Given the time required to accrue enough patients to reach statistically definitive conclusions combined with the important facts that lower MED together with an encouraging reduction in AE2+ were easily achieved without degrading target coverage or disrupting clinical workflow, we are continuing to use the new MED guidelines for future patients.

## 5 Conclusion

To reduce the risk of esophageal toxicity in NSCLC patients receiving conventionally fractionated radiation therapy (2 Gy/fraction for approximately 30 fractions) planners were requested to try to achieve a MED below 21 Gy if possible while still respecting target coverage and other OAR constraints. The requested MED was based on a formal outcomes study of a large group of NSCLC patients (3,4). If an otherwise acceptable treatment plan could not be achieved with such a low MED, planners were instructed to aim for a MED between 21 Gy and the prior planning goal of 34 Gy. Reducing the MED was not a mandatory ‘hard constraint’, but rather intended to be a ‘gentle reminder’. The plans for patients one year before (Group A) and one year after (Group B) this change were analyzed. A statistically significant MED reduction was accomplished. Although the reported incidence of AE2+ was lower in Group B, this was not statistically significant. Nevertheless, the clinically observed overall incidence of AE2+ was significantly lower in patients with mean doses below 21 Gy and there was good agreement between the observed rates of esophagitis and those predicted by the model both before and after the guideline was implemented. Implementing ‘soft’ planning guidelines to better control normal tissue doses is feasible and can improve clinical outcomes.

## Authors’ Contributions

E. Yorke designed the study, reviewed the patients for relevance, collected the patient‐specific data, did some of the data analysis and did the initial drafting of the manuscript. M. Thor and A. Jackson performed data analysis and contributed to drafting the manuscript, particularly in regard to the data analysis. D. Gelblum, D. Gomez, A. Rimner, N. Shaverdian, A. Shepherd, C. B. Simone, and A. Wu agreed to use the new planning guidelines for their patients, performed ‘on treatment visit’ reviews during their patients’ treatment courses and contributed to the drafting of the manuscript particularly in regard to the medical implications of the esophagitis guideline. D. McKnight identified candidate patients from the department records. All authors read the manuscript and agreed to the submission.
